# Reflection on commentary “Miles to go before I seek: distance to the health facility and health care use among older adults in India”

**DOI:** 10.1016/j.lansea.2025.100679

**Published:** 2025-10-04

**Authors:** Sanjay K. Mohanty, Abhishek Kumar, Paul Weiss

**Affiliations:** a Department of Population & Development, International Institute for Population Sciences, Mumbai, India; b College of Social Science, University of Lincoln, London, England

## Reflection on distance to the health facility and health care use among older adults in India

A recent commentary entitled “*Miles to go before I seek: distance to the health facility and health care use among older adults in India”* published in “*The Lancet South Asian Studies”* by Dr Misra and colleagues highlighted distance to health facilities as a barrier in accessing the outpatient and inpatient services among elderly in India.^1^ The distance travelled to access health care services depends on multiple factors, including availability of services, transportation facilities, type of diseases, severity of diseases, income of the household, fees charged and preferences of the patients,^1^, ^2^, ^3^, ^4^ that may call for a comprehensive analysis.

The commentary by Mishra et al. (2025) is a thought-provoking description and used an appropriate data set in the Indian context. The main inferences drawn from the commentary suggest distance as an inhibitor in the use of health services. The impact of this publication is strong, as the issue was raised in the upper house of the Indian parliament (Rajya Sabha) recently. While the conclusions are appealing, we present the alternative averages that are best suited for skewed distributions and the estimated distance travelled by a specific disease. We also observed that the interpretation of the given table in the commentary needs to be made differently.

We analysed the sub-sample of 31,902 individuals 60 years and above, who were successfully interviewed in the Longitudinal Ageing Study in India (LASI) Wave-1 (2017–18).^5^ The distance was computed among respondents who reported use of outpatient or inpatient services from a health centre in the 12 months prior to the survey. As the distances travelled (km) were right-skewed, we prefer to report the median distance, the geometric mean (GM) with 95% confidence intervals (CI) and the geometric standard deviation (GSD). We have also computed the coefficient of variation (CV) to understand the relative variability. We have used the survey design (strata, primary sampling units, and probability weights) in the analysis. GMs and CIs were obtained by estimating the mean and standard error of the log of distance travelled and back-transforming; GSD was computed using the standard deviation of the log of distance and back-transformed. As there were zero values in the data set, we used log (distance+ 1) and subtracted 1 after back-transformation.

We emphasise that the median is the preferred descriptive indicator of the typical travel burden given skewness in the distribution. However, the median lacks straightforward sampling distributions for survey inference. To address this, we additionally report the geometric mean (GM) and geometric standard deviation (GSD), which mitigate the influence of extreme outliers while preserving the inferential advantages of mean-based statistics. Thus, GM lies between AM and Median (closer) and contains both interpretability near the median and inferential tractability like the mean. To visualise distributional differences across disease categories, we used boxplots that display the median and interquartile range. The analyses were carried out in Stata 17.

We presented the histogram of distance travelled for both outpatient and inpatient services (Figures S1 and S2) for rural and urban areas. It may be mentioned that the distance travelled had a skewness coefficient of 22.0 and 10.5 for outpatient and inpatient care, respectively (Table 1). The arithmetic means are more likely to be affected by extreme observations in both OPD/IPD services. Mishra and colleagues estimated the arithmetic mean of distance for outpatient services at 14.5 km and 43.6 km for inpatient services. We estimated the median distance at 4 km (Table 1) for outpatient services and 15 km for inpatient services. We also provided the geometric means, which were closer to the medians across settings, corroborating the estimates. For example, among rural inpatients, the GM (95% CI) was 23.0 (20.7–25.6) km versus a median of 20 km and an arithmetic mean of 51.2 km, indicating that extreme transfers inflate the mean. The pattern was similar in the case of urban areas as well. The inferences drawn by using the median and GM are similar for inpatient and outpatient services across rural and urban areas (Table 1). The coefficient of variation analysis shows that there is more relative variability in urban settings (CV = 6.5) compared to rural settings (CV = 2.6) in the case of outpatient care distance.

**Table 1:** Arithmetic mean, geometric mean and median distance travelled (in KM) to avail outpatient and inpatient services among the elderly in India, 2017–18.

Category	Arithmetic mean of distance travelled (km)	Coefficient of variation (CV)	Median distance (km)	Geometric mean of distance travelled (95% CI)	Geometric standard deviation of distance travelled	Skewness	N
**Outpatient**							
Rural	15.5	2.6	5	6.0 (5.6–6.4)	3.5	15.3	10,851
Urban	12.4	6.5	2	3.1 (2.9–3.4)	3.1	19.6	6028
Combined	14.5	3.8	4	5.0 (4.7–5.2)	3.5	22.0	16,879
**Inpatient (Hospitalisation)**							
Rural	51.2	2.3	20	23.0 (20.7–25.6)	3.3	10.4	1601
Urban	27.1	2.9	6	7.9 (6.5–9.5)	3.7	8.5	894
Combined	43.6	2.5	15	16.5 (15.0–18.3)	3.7	10.5	2495

GSD, Geometric Standard Deviation of distance travelled, a measure of relative variability on a multiplicative scale.

We also observed that authors in their commentary presented the distribution of distance for outpatient and inpatient services in varying categories. The interpretation provided in their table may not be accurate. It may be mentioned that the question on distance travelled to the most recent outpatient visit was asked only to those individuals who availed of the outpatient/inpatient services. The table published in the commentary is the distribution of the sample by distance coverage. It may be interpreted that among those who availed outpatient services, 73.1% travelled 0–10 km, 17.1% travelled 11–30 km, 5.5% travelled 31–60 km, and 3.3% travelled over 60 km. This is different from the care utilisation rate as explained in table. A similar interpretation may be made for inpatient services. Thus, interpreting the sample distribution as the prevalence of outpatient/inpatient services is not correct.

**Fig. 1:**
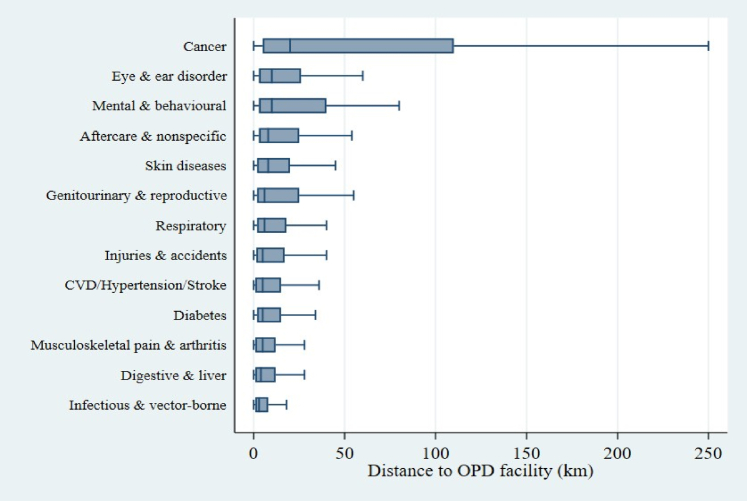
**Distribution of distance travelled for outpatient care across major disease categories. Boxplots display the interquartile range (IQR), median (horizontal line), and adjacent values (whiskers). Outliers are not shown for clarity**.

**Fig. 2:**
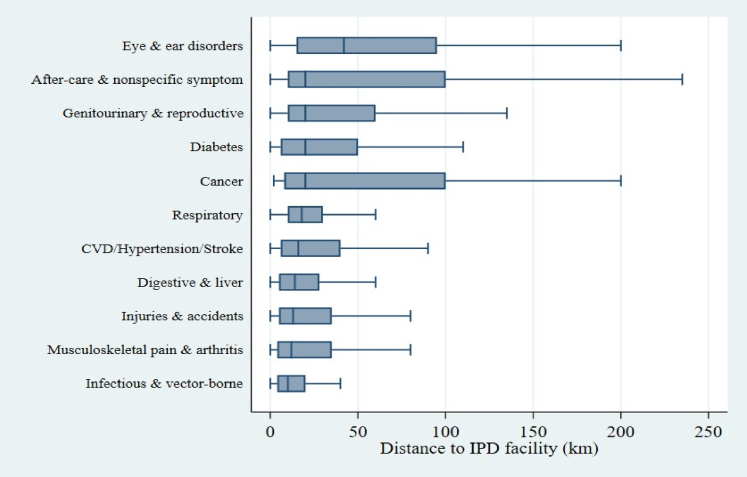
**Distribution of distance travelled for inpatient care across major disease categories. Boxplots display the interquartile range (IQR), median (horizontal line), and adjacent values (whiskers). Outliers are not shown for clarity**.

The distances travelled vary greatly by disease treated and other socio-economic conditions. The availability of super-speciality services for certain diseases, such as cancer, heart disease, and mental disorders, is not available in small towns or urban areas. Our analyses suggest that the distance travelled for cancer treatment was highest, followed by eye and ear-related disorder and mental and behavioural disorder for outpatient services (Fig. 1, Table S1) and highest for eye and ear disorder, followed by cancer, aftercare and diabetes in case of inpatient care utilisation (Fig. 2, Table S2).

We acknowledged that the distance covered was reported by respondents and may be subject to reporting bias. To sum up, we agree that distance is an inhibitor in accessing health care services among the elderly in India. However, the use of appropriate statistical methods and comprehensive analyses may provide insight into the accessibility of health services. As the arithmetic mean exaggerates central tendency, while the median—though robust—has less straightforward sampling distributions under survey design. GM is closer to the physical center of the distribution than the arithmetic mean, yet allows for standard inferential procedures (standard errors, confidence intervals) using survey-weighted log transformations. This dual approach enhances both descriptive clarity and statistical rigour to the analysis. Finally, the coefficient of variation standardised the variability, providing better estimates for urban-rural differences in relative variability in accessing healthcare.

## Contributors

The study was conceptualised by SKM. The data curation and formal analysis were done by AK. The first draft was prepared by SKM and AK. PW reviewed the statistical approach and contributed to the refinement of the analysis and manuscript revision. This was followed by editing and the writing of the final draft by AK & SKM. (SKM: Sanjay K Mohanty, AK: Abhishek Kumar, PW: Paul Weiss).

## Data sharing statement

SKM and AK have full access to the data. Sharing of the data with outside parties is at the discretion of the corresponding author and may be considered on request.

## Data availability statement

Data used in this correspondence were taken from the publicly available data source that can be accessed on https://www.iipsindia.ac.in/content/LASI-data. The codes for analysis are available upon request from the authors.

## Declaration of interests

The authors declare no competing interests.
