# Supplementary material for: Reflection on commentary “Miles to go before I seek: distance to the health facility and health care use among older adults in India”
Source: Lancet Reg Health Southeast Asia. 2025 Oct 4;45:100679. doi: 10.1016/j.lansea.2025.100679 (PMC12926033; doi:10.1016/j.lansea.2025.100679)
Supplement: Supplementary Figures and Tables [file mmc1.docx]

**Reflection on commentary “Miles to go before I seek: distance to the health facility and health care use among older adults in India”**

**Supplementary Material (SM)**

**Table of contents**

Figure S1. Distribution of Distance travelled for outpatient care among the elderly in India, 2017-18. ………………………………………………………………………………………….........................2

Figure S2. Distribution of Distance travelled for inpatient care among the elderly in India, 2017-18...2

Table S1. Disease-specific variations in average distance travelled (in kilometres) among the elderly for outpatient care in India, 2017-18. …………………………………………………3

Table S2. Disease-specific variations in average distance travelled (in kilometres) among the elderly to access inpatient care in India, 2017-18 ……………............... .................................3


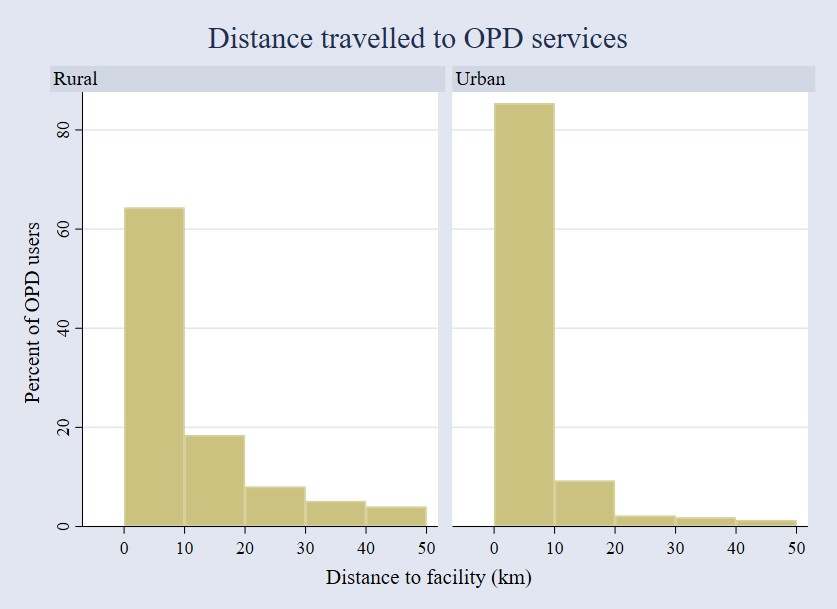


**Figure S1. Distribution of Distance travelled for outpatient care among the elderly in India, 2017-18.**


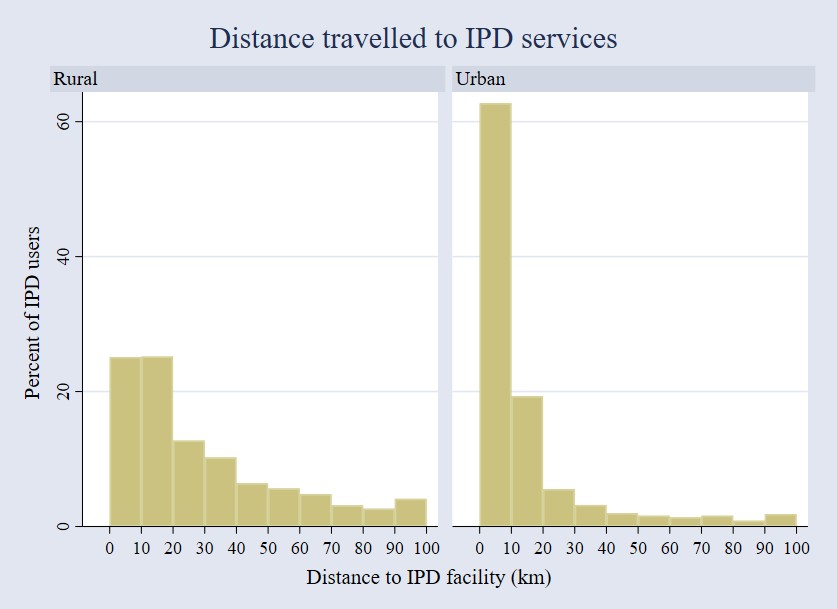


**Figure S2. Distribution of Distance travelled for inpatient care among the elderly in India, 2017-18.**

**Table S1. Disease-specific variations in average distance travelled (in kilometres) among the elderly for outpatient care in India, 2017-18.**

| **Disease groups** | **Arithmetic Mean of distance travelled (km)** | **Coefficient of variation (CV)** | **Median distance travelled (km)** | **Geometric Mean of distance travelled (95% CI)** | **Geometric Standard Deviation of distance travelled (GSD)** | **N** |
| --- | --- | --- | --- | --- | --- | --- |
| Cancer | 247.0 | 2.2 | 20 | 31.1 (11.4–81.6) | 7.8 | 63 |
| Musculoskeletal Pain & Arthritis | 14.3 | 4.4 | 5 | 5.0 (4.5–5.4) | 3.3 | 3569 |
| Infectious & Vector Borne | 8.5 | 3.2 | 3 | 3.3 (3.0–3.6) | 3.1 | 4183 |
| Mental & Behavioural | 36.3 | 2.7 | 10 | 10.9 (7.1–16.4) | 4.4 | 119 |
| Diabetes | 18.9 | 3.3 | 5 | 6.5 (5.8–7.3) | 3.5 | 1452 |
| CVD/Hypertension/Heart–Stroke | 17.9 | 3.0 | 5 | 5.7 (5.0–6.3) | 3.7 | 2649 |
| Chronic Respiratory (Asthma/COPD) | 14.7 | 1.6 | 6 | 6.5 (5.5–7.8) | 3.4 | 625 |
| Digestive & Liver | 12.5 | 2.2 | 4 | 4.8 (4.3–5.4) | 3.4 | 1141 |
| Injuries & Accidents | 13.6 | 1.5 | 5 | 5.8 (4.6–7.4) | 3.5 | 472 |
| Eye & Ear Disorder | 24.3 | 2.1 | 10 | 9.5 (8.1–11.3) | 3.7 | 622 |
| Genitourinary & Reproductive | 24.6 | 2.6 | 6 | 8.0 (5.5–11.3) | 4.2 | 183 |
| Skin Diseases | 18.6 | 1.6 | 8 | 7.6 (6.0–9.6) | 3.7 | 264 |
| Aftercare & Nonspecific | 26.4 | 3.0 | 8 | 8.9 (7.1–11.1) | 3.7 | 299 |

*GSD = Geometric Standard Deviation of distance travelled is a measure of relative variability on a multiplicative scale.*

**Table S2. Disease-specific variations in average distance travelled (in kilometres) among the elderly to access inpatient care in India, 2017-18.**

| **Disease groups** | **Arithmetic Mean of distance travelled (km)** | **Coefficient of variation (CV)** | **Median distance travelled (km)** | **Geometric Mean of distance travelled (95% CI)** | **Geometric Standard Deviation of distance travelled (GSD)** | **N** |
| --- | --- | --- | --- | --- | --- | --- |
| Cancer | 93.9 | 2.2 | 20 | 28.3 (13.9–57.0) | 4.7 | 35 |
| Musculoskeletal Pain & Arthritis | 29.2 | 1.4 | 12 | 13.5 (9.8–18.5) | 3.4 | 178 |
| Infectious & Vector Borne | 28.7 | 3.4 | 10 | 10.7 (8.3–13.9) | 3.3 | 400 |
| Diabetes | 45.4 | 1.4 | 20 | 18.9 (13.1–27.0) | 3.9 | 146 |
| CVD/Hypertension/Heart–Stroke | 61.3 | 2.9 | 16 | 19.1 (15.1–24.2) | 4 | 480 |
| Chronic Respiratory (Asthma/COPD) | 34.5 | 2.0 | 18 | 16.6 (13.0–21.1) | 3.2 | 176 |
| Digestive & Liver | 27.7 | 2.4 | 14 | 12.6 (10.4–15.3) | 3.2 | 375 |
| Injuries & Accidents | 36.4 | 2.2 | 13 | 14.1 (10.7–18.3) | 3.7 | 241 |
| Eye & Ear Disorder | 69.4 | 1.4 | 42 | 35.9 (27.4–46.9) | 3.5 | 161 |
| Genitourinary & Reproductive | 55.6 | 1.7 | 20 | 23.7 (17.4–32.2) | 3.6 | 110 |
| Aftercare & Nonspecific | 62.6 | 1.3 | 20 | 24.2 (14.8–39.2) | 4.5 | 84 |

*GSD = Geometric Standard Deviation of distance travelled is a measure of relative variability on a multiplicative scale.*
